# “ShapeNet”: A Shape Regression Convolutional Neural Network Ensemble Applied to the Segmentation of the Left Ventricle in Echocardiography

**DOI:** 10.3390/jimaging11050165

**Published:** 2025-05-20

**Authors:** Eduardo Galicia Gómez, Fabián Torres-Robles, Jorge Perez-Gonzalez, Fernando Arámbula Cosío

**Affiliations:** 1 Instituto de Investigaciones en Matemáticas Aplicadas y en Sistemas, Universidad Nacional Autónoma de México, Mexico City 04510, Mexico; gagoed@comunidad.unam.mx; 2 Laboratorio de Física Médica, Instituto de Física, Universidad Nacional Autónoma de México, Mexico City 04510, Mexico; ftrobles@fisica.unam.mx; 3 Unidad Académica del Instituto de Investigaciones en Matemáticas Aplicadas y en Sistemas en Yucatán, Universidad Nacional Autónoma de México, Merida 97357, Mexico; jorge.perez@iimas.unam.mx

**Keywords:** active shape models, convolutional neural networks, echocardiography, left ventricle segmentation, shape constraints, ultrasound segmentation

## Abstract

Left ventricle (LV) segmentation is crucial for cardiac diagnosis but remains challenging in echocardiography. We present ShapeNet, a fully automatic method combining a convolutional neural network (CNN) ensemble with an improved active shape model (ASM). ShapeNet predicts optimal pose (rotation, translation, and scale) and shape parameters, which are refined using the improved ASM. The ASM optimizes an objective function constructed from gray-level profiles concatenated into a single contour appearance vector. The model was trained on 4800 augmented CAMUS images and tested on both CAMUS and EchoNet databases. It achieved a Dice coefficient of 0.87 and a Hausdorff Distance (HD) of 4.08 pixels on CAMUS, and a Dice coefficient of 0.81 with an HD of 10.21 pixels on EchoNet, demonstrating robust performance across datasets. These results highlight the improved accuracy in HD compared to previous semantic and shape-based segmentation methods by generating statistically valid LV contours from ultrasound images.

## 1. Introduction

According to the Centers for Disease Control and Prevention (CDC) in the United States [1], one in four individuals with heart failure die each year, with most cases linked to dysfunction in the left ventricle (LV). The LV plays a crucial role in distributing oxygenated blood throughout the body via the aortic valve. Any malfunction in this process can lead to severe complications within the circulatory system and other organs. As reported by Berman et al. [1], heart failure often results from compromised LV function, typically due to structural changes in the ventricular wall or the inability of the LV to fill or eject blood effectively. Patients with cardiac disease frequently experience symptoms such as dyspnea, fatigue, and fluid retention, which can further progress to ischemia, muscle disease, pulmonary congestion, and elevated heart rate. To assess ventricular function, a range of imaging and signal processing methods are currently available, including physical examination, X-rays, electrocardiogram (ECG), magnetic resonance imaging (MRI), and echocardiography (ultrasound). Among these, echocardiography provides valuable insights into both systolic and diastolic LV function, ventricular morphology, and conditions such as aneurysms, along with mitral, tricuspid, aortic, and pulmonary valve function [2]. Due to its non-invasive nature and excellent cost–benefit ratio, echocardiography is widely used in clinical practice for evaluating ventricular function [2].

However, accurately defining the LV contour and shape remains a critical challenge for diagnosing heart failure. Computational methods have emerged to support cardiologists in producing more precise and efficient diagnoses. Currently, deep-learning-based approaches, particularly those employing semantic segmentation like convolutional neural networks (CNNs), have shown promising results. Nevertheless, these methods can produce anatomically inconsistent or noisy LV contours, including implausible segmentations with irregular or disconnected regions that do not correspond to the expected LV morphology. Such artifacts, commonly referred to as *blobs* in medical image analysis, result from pixel-level misclassification errors inherent in semantic segmentation approaches and may significantly compromise clinical reliability. In this work, we address these limitations by presenting a new method for LV segmentation, and the novelty of this work lies in three key contributions. First, we introduce ShapeNet, a specialized ensemble of CNNs that directly predicts both the pose parameters (rotation, translation, and scale) and shape deformation parameters of a statistical shape model, eliminating the artifacts produced by pixel misclassification in semantic segmentation methods (blobs). Second, we develop an improved ASM that optimizes a global objective function based on concatenated gray-level profiles, demonstrating superior capture range and robustness compared to traditional ASM approaches. Third, our fully automatic pipeline uniquely combines these components to generate anatomically plausible contours, without manual initialization—a significant advantage over semi-automatic methods like BEASM [3]. This integrated approach maintains the flexibility of data-driven deep learning, while utilizing the anatomical validity offered by shape models, as evidenced by our consistent performance across both CAMUS and independent EchoNet datasets.

## 2. Related Work

This section provides an overview of the key methods in LV segmentation, highlighting their challenges and how our proposed method addresses these limitations.

### 2.1. Deep Learning Approaches

Recent developments and applications in CNNs [4,5,6] have led to significant progress in the automatic segmentation of organs across various medical imaging modalities. CNNs have become a commonly used method for segmenting the region of interest (ROI), followed by annotation of the boundary. For example, Chen et al. [7] reviewed prominent deep-learning techniques, highlighting that U-Net and its variants are widely used in medical image segmentation tasks, including ensembles of different architectures of CNNs and transformer networks for organ region detection or using mask region CNNs which produce a bounding box and a binary mask of the organ. In [8,9,10], the U-Net architecture was successfully applied to the classification of pixels corresponding to the LV. Ansari et al. [11] proposed a novel CNN based on a U-Net backbone with PSP in the skip connections. A thorough evaluation was performed to assess the benefits of preprocessing with a contrast limited adaptive histogram equalization (CLAHE), showing improved results for real-time segmentation of ultrasound videos of the liver. Kang et al. [12] reported a new CNN architecture for the segmentation of the LV in transesophageal ultrasound taken during cardiopulmonary resuscitation procedures (CPR). The CNN includes an attention mechanism and a residual feature aggregation module able to accurately segment the LV in the presence of large shadows and atypical deformations. Zhao et al. [13] reported a semi-supervised echocardiography semantic segmentation method, which is able to segment the left ventricle, epicardium, and left atrium on ultrasound images. The method is based on a boundary attention transformer net and a multi-task semi-supervised model with consistency constraints. This approach enables effective model training with a partially annotated training set. In addition, Shi et al. [14] proposed a hybrid transformer–CNN architecture to enhance segmentation robustness, combining ResNet-50 for spatial feature extraction with a transformer-based encoder–decoder for global context modeling. Their framework integrates two key modules: a Convolutional Block Attention Module (CBAM) to adaptively fuse CNN and transformer features, enhancing focus on anatomically relevant regions, and a Bridge Attention (BA) mechanism to filter non-relevant features, while refining segmentation maps through multi-level feature aggregation. While CNNs have revolutionized segmentation tasks, these methods face several challenges, especially in applications like echocardiography, where the LV boundaries can be imprecise or noisy. Despite their ability to learn spatial features from large datasets, CNNs tend to produce anatomically inconsistent contours when dealing with variations in heart shape, speckle noise, and imaging artifacts.

### 2.2. Traditional Machine Learning Methods

Previous machine learning approaches for LV segmentation, including geodesic models [15], level sets [16], and shape-based deformable models [17], offer effective ways to model and deform contours based on anatomical points. Moreover, Statistical Shape Models (SSMs) have been particularly effective in left ventricle segmentation when closely initialized [18]. SSMs provide an effective means to incorporate expert shape knowledge into organ segmentation techniques. These methods rely on predefined shape models and iterative algorithms to find the optimal boundary. However, they often require accurate initialization and are sensitive to the quality of the input data, which makes them less robust when dealing with noisy or low-quality images. Registration-based techniques [19], supervised learning [20], and active appearance models [21] have also been explored for LV segmentation, in combination with the availability of large annotated datasets [22]. These methods integrate image registration with statistical models to align anatomical shapes. While they provide more accurate segmentation in some cases, they are often computationally expensive and require manual intervention for initialization, making them less practical for routine clinical use. Despite their strengths, these approaches have limitations in terms of robustness, scalability, and flexibility, particularly in dealing with complex, real-world datasets like echocardiography images.

### 2.3. Hybrid Methods

Hybrid methods that combine SSMs, ASMs, and CNNs have gained attention as a way to leverage the strengths of both paradigms. For example, Li et al. [23] introduced a hybrid method where a CNN first detects three key landmarks on the LV: the apex and the starting and end points of the endocardium. These landmarks are used to initialize a deep-snake model [24], which then adjusts the contour using circular convolution. This method demonstrated strong performance on the HMC-QU echocardiography dataset, but its reliance on landmark-based initialization limits its flexibility in more challenging scenarios. Hybrid methods combining region-based CNNs and ASMs have also been explored. Wei-Yen et al. [25] proposed a method where a CNN detects a bounding box around the LV, which is then used to initialize the ASM for final contour refinement. While this approach is promising, it still requires accurate initialization and may struggle with complex deformations. Our approach builds on these hybrid techniques by combining a CNN ensemble (ShapeNet) with a Point Distribution Model (PDM), ensuring fully automatic initialization and the generation of anatomically plausible contours. This integration enhances robustness by eliminating the need for manual intervention, unlike previous hybrid methods.

### 2.4. Methods Incorporating Anatomical Constraints

In recent years, methods incorporating anatomical constraints have shown improved performance for LV segmentation. Oktay et al. [26] developed the Anatomically Constrained Neural Network (ACNN), which combines a CNN with an autoencoder trained on ground truth ventricle masks. Anatomical constraints are included during training of the objective function, which is made of a linear combination of cross entropy, shape regularization, and weight decay terms. Shape regularization is implemented with a distance function between the encoded ground truth mask and the encoded prediction of the segmentation CNN. The ACNN architecture was evaluated on both MRI and ultrasound images, demonstrating improved performance over previous approaches.

Gaggion et al. [27] proposed a hybrid CNN architecture that uses graph convolutional networks to impose anatomical constraints on the latent space. This method integrates image-based feature extraction with landmark-based shape modeling, effectively ensuring anatomically valid segmentations. The model is trained with pairs of input images and the corresponding landmark annotations of the organs of interest, the same number of landmarks is used in all the training examples. This approach was validated on chest X-ray images and demonstrated favorable results for anatomical structure segmentation.

Ribeiro et al. [28] presented a fully-automatic hybrid method for LV segmentation in cardiac MRI images, combining deep learning and deformable models. Initially, a ROI containing the LV is extracted using heart movement analysis. A deep learning network (DLN) is then employed to generate an initial segmentation of the LV cavity and myocardium. DLN-based segmentation is subsequently used to estimate exam-specific statistical information about the LV, which helps initialize and constrain a level-set-based deformable model. This deformable model incorporates anatomical constraints to refine the segmentation and generate the final result. In the final step, failed segmentations are detected and corrected using information from adjacent frames. We also reported a modified U-Net in a previous work [29], with a regression layer replacing the final classification layer, enabling the model to predict LV pose and shape parameters directly.

While deep learning methods, traditional shape-based techniques, and hybrid approaches have made significant strides in LV segmentation, each faces limitations related to initialization, anatomical accuracy, and robustness across diverse datasets. Our proposed method addresses these challenges by combining a CNN ensemble with a PDM, providing a fully automated, anatomically statistically consistent, and robust solution for LV segmentation.

## 3. Materials and Methods

**Figure 1:**
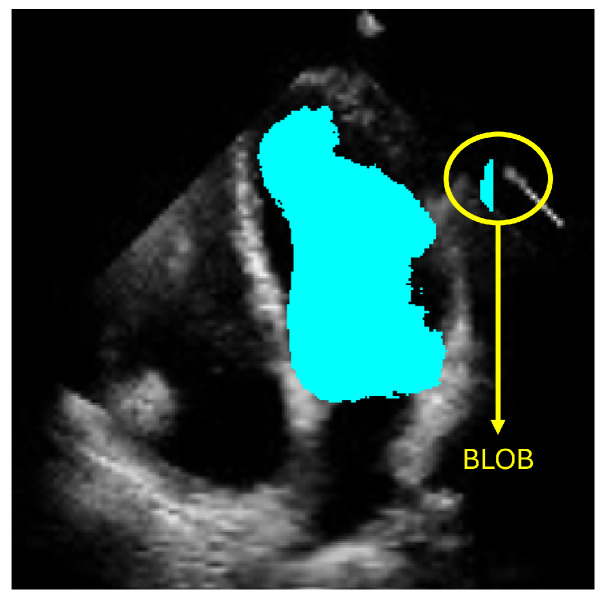
Example of blobs produced by misclassification in semantic segmentation of the left ventricle.

The objective of ShapeNet is the accurate characterization the left ventricle contour through the prediction of optimal pose (rotation, translation, and scale) and shape (deformation vector) parameters for a trained point distribution model (PDM) of the LV in echocardiography. This is achieved by training a CNN with the aforementioned parameters of the PDM that have been accurately adjusted to the left ventricle on each image of the training set. This approach prevents the formation of blobs produced by pixel classification errors in semantic classification, preventing correct characterization of the LV, as illustrated in Figure 1. In contrast, our approach consistently produces statistically valid contours of the left ventricle, which are accurately adjusted by means of the improved ASM proposed in this paper.

### 3.1. Point Distribution Model of the Left Ventricle (LV-PDM)

**Figure 2:**
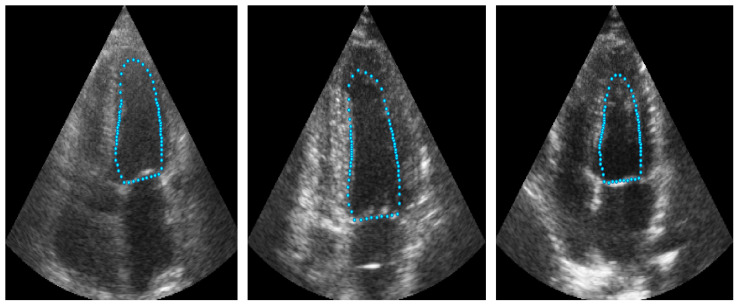
Example of landmarks sampled in LV ultrasound images.

**Figure 3:**
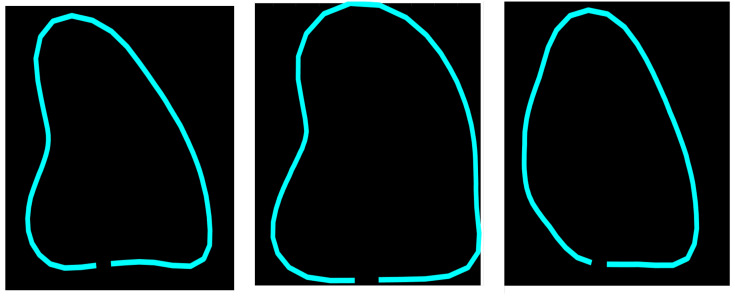
Example of different shapes of the LV using the principal variation modes calculated from the PDM.

Point distribution models (PDMs) provide a compact representation of a class of shapes; in this case, the shape of the left ventricle. Construction of the PDM was performed as described in [30] by annotating landmark points around the contour of each left ventricle previously marked by an expert. For this LV-PDM, 64 landmarks were selected, which accurately represent the contour of the left ventricle, as shown in Figure 2, using the training data described in Section 4.1. Principal component analysis (PCA) of the normalized landmark training set resulted in five principal modes of variation in the shape of the LV, contained in a principal eigenvector matrix (ϕ), which allows creating new instances of the LV shape $\hat{s}$ using Equation (1). Figure 3 shows some examples of shapes, corresponding to different values of the five weights in vector *b*.

(1) $$\hat{s}=\bar{s}+\phi b$$

where

$\hat{s}$ = LV shape.
$\bar{s}$ = the mean shape of the training set.
ϕ = principal eigenvector matrix of the training set.
*b* = vector of deformation parameters of the training set.

The final contour of a ventricle (*R_Shape*) on an echocardiography is defined using Equation (2).

(2) $$R\_Shape=\sigma \left[\begin{array}{cc} cos(\theta ) & -sin(\theta )\\ sin(\theta ) & cos(\theta ) \end{array}\right]\hat{s}+\left[\begin{array}{c} T_{x},T_{y} \end{array}\right]$$

where

R_Shape = reconstructed LV shape on echocardiography.
$\hat{s}$ = shape obtained after applying Equation (1).
σ = the scale of the LV.
θ = the rotation of the LV.
Tx = translation in X axis of the LV.
Ty = translation in Y axis of the LV.

ShapeNet was trained to predict all the pose $\left(\sigma ,\theta ,T_{x},T_{y}\right)$ and shape parameters (b), as described below.

### 3.2. ShapeNet Architecture and Training

The architecture is inspired by encoder–decoder architectures, taking as a basis the encoder part and its power for image feature extraction and dimensionality reduction, hence the origin of the first block. Then, a second block formed by fully connected layers and a regression layer are used to relate the features extracted in the first block with the pose and shape values extracted from the PDM. This architecture is designed to be used and implemented on off-the-shelf computing equipment. The two blocks mentioned above and the full architecture are detailed below.

**Figure 4:**
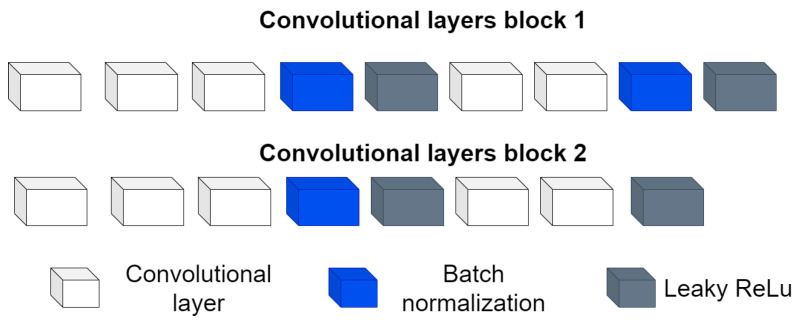
Structure of the convolutional block.

**Figure 5:**
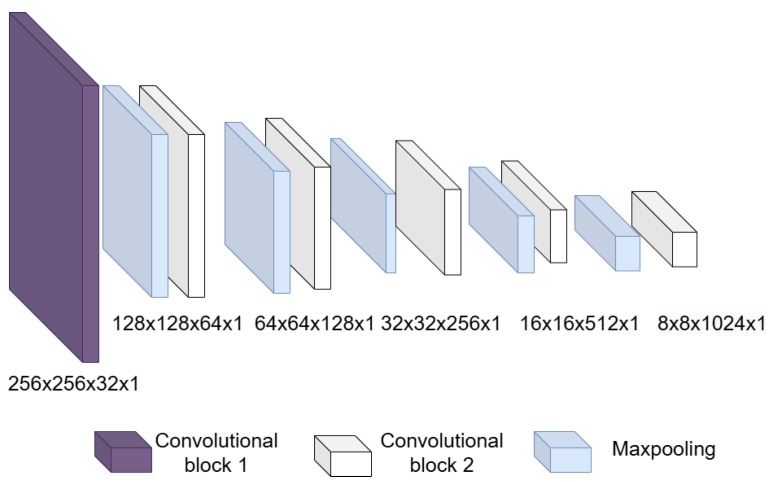
Overview of the convolutional section of ShapeNet.

**Figure 6:**
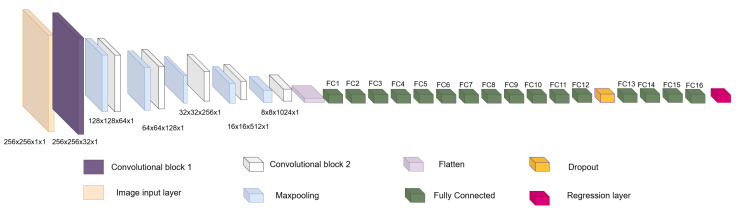
Overview of ShapeNet architecture.

1. Input layer: This is an image input layer of size 256 × 256 × 1 of the form: (width, height, channels). This is an echocardiographic image of the left ventricle.
2. Convolutional block: This is formed of 2 parts, as shown in Figure 4, the convolution filters are filters of size 3 × 3 with a stride of 1, and the number of filters increases, as shown in Figure 5, starting with 32 filters and doubling the number at each convolution stage. The purpose of these convolutional layers is to extract features from the LV image, which will later be used to link them with the pose and shape features extracted from the previously trained LV-PDM. In addition, there are maxpooling layers in between each convolution stage with a stride of 2; with maxpooling, the dimensionality of the problem is reduced and the most important features of the image are preserved. Furthermore, this allows the training process to be lighter, since it reduces the number of parameters that have to be learned by ShapeNet. An overview of this block is shown in Figure 5.
3. Fully connected block: This consists of a flatten layer followed by a set of fully connected and one dropout layer, to avoid data over-fitting during training. This block links the features extracted in the convolutional block with the pose and shape parameters of the left ventricle and then adjusts the weights to make predictions in the regression layer. Finally, at the end of the fully connected block, a regression layer (Figure 6 pink block) calculates one, pose or shape, parameter: rotation, translation, scale, or $b_{i}$ for a given input image.

In order to train ShapeNet, the pose parameters rotation (θ), translation (Tx,Ty), and scale (σ), as well as the shape parameters *b*, were extracted from each LV contour example in the training set as follows:

- Translation (Tx,Ty): The translation was calculated as the mean of the LV contour coordinates on the X axis and Y axis for each example in the training set.
- Rotation (θ): This value was calculated using the binary mask of each example, enclosing it within an ellipse and then calculating the angle between the X axis and the major axis of the ellipse using the second moments of the mask.
- Scale (σ): This was calculated as shown in [30] by normalizing each LV shape to a common scale and minimizing the root-mean-square distance between the corresponding landmarks of the LV *i*-th training shape and the mean shape $\left(\bar{s}\right)$ obtained in Section 3.1.
- Shape parameters (*b*): The deformation parameters were extracted from the expert annotation by solving for the value of *b* in Equation (1) for each contour in the training set, as shown in Equation (3).

(3) $$b=\left({\hat{s}}_{i}-\bar{s}\right)*\phi^{\prime }$$

where

${\hat{s}}_{i}$ = The expert annotation of the *i*-th example in the training set.
$\bar{s}$ = The mean LV shape of the training set.
$\phi^{\prime }$ = The transposed principal eigenvector matrix of the training set.

After extracting the aforementioned parameters of the LV shape, a training vector (V) was constructed with the shape and pose parameters of the left ventricle in the corresponding training image, as shown in Equation (4).

(4) $$V=[I_{i},\beta_{i}]$$

where

$I_{i}$ = the *i*-th image of the training set.
$\beta_{i}$ = Is the corresponding shape or pose parameter to be learned by the network.

Our approach is based on an ensemble of networks, where each network is trained to optimize one specific parameter of pose and shape of the left ventricle; therefore, we developed dedicated networks for rotation (θ), translation along both the X ($T_{x}$) and Y ($T_{y}$) axes, scale (σ), and each of the five deformation parameters contained in vector *b* in Equation (1). Consequently, our method involves the training of nine networks, one for each pose $\left(\sigma ,\theta ,T_{x},T_{y}\right)$ and shape parameter (b).

During the training of each network, a vector *V* is used as input, depending on which parameter β needs be trained. Consequently, the network adjusts the weights according to a loss function defined as the root mean squared error (RMSE) between the value predicted by the network ($\beta_{p}$) and the parameter $\beta_{i}$ contained in the input vector *V* (see Equation (5)).

(5) $$RMSE=\sqrt{\frac{\sum_{i=1}^{n}{\left|\beta_{p}-\beta_{i}\right|}^{2}}{n}}$$

where:

$\beta_{p}$ = Value predicted by the network.
$\beta_{i}$ = Input value contained in the *V* vector.
*n* = The number of images in the training set.

Additionally, other important hyperparameters to consider during our network training are the batch size and learning rate. For this paper, a batch size of 64 images was used, which we consider to be a moderate size and manageable by most modern GPUs. Moreover, as mentioned by [31], increasing the batch size does not have a significant impact on the accuracy of the gradient calculation when using the ADAM optimizer (which was employed to train these networks). Regarding the learning rate, a rate of $1\times 10^{-3}$ was used, as we consider this to be not too high to destabilize training, and not too low to slow down convergence. This leads us to the convergence criterion, which was early stopping. According to our experiments, most networks concluded with an average of 50 epochs; beyond this number of epochs, the RMSE value for validation did not change. The validation patience parameter was set to 40 iterations.

After the *ShapeNet* has been trained as described above, the next step involves an improved ASM, as detailed in the next section.

### 3.3. Improved ASM

Following the automatic initialization of the LV-PDM using ShapeNet, an active shape model was used to improve the segmentation accuracy. This ASM is based on the optimization of an objective function constructed with a set of gray-level profiles sampled around the contour of the left ventricle in an echocardiogram. Gaussian filtering (σ = 0.8, 3 × 3 kernel) was applied to reduce speckle noise, while preserving edge information of the ultrasound images, before adjusting the ASM, as described in [32].

#### ASM Objective Function

**Figure 7:**
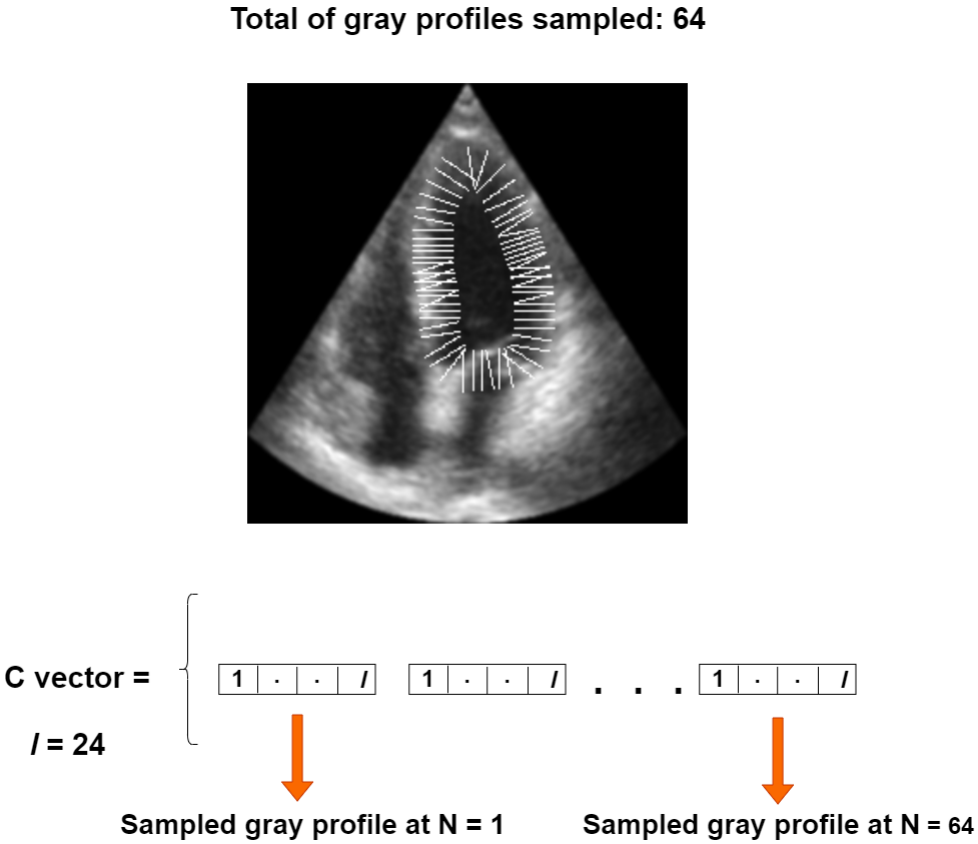
Construction of the *C* vector for one training instance.

Our objective function is based on a set of perpendicular gray level profiles of length *l*, sampled from each landmark point in an image of the training set, as proposed in [30]. All gray profiles are concatenated into a single vector, referred to as the *C* vector, with a length of 64×l for our 64-point LV-PDM, as illustrated in Figure 7.

The mean vector of the training set ($\bar{C}$) is given by Equation (6), while the objective function ($f_{C}$) represents the RMSE distance between $\bar{C}$ and a newly sampled vector $\left(C_{new}\right)$ from a new echocardiogram, as shown in Equation (7). During image segmentation, f(c) is optimized by iteratively alternating the local search of the ASM [30] with optimization using the simplex algorithm, as previously reported in [32].

(6) $$\bar{C}=\frac{\sum_{i=1}^{n}C_{i}}{n}$$

where

n = number of examples in the training set.
$C_{i}$ = the *i*-th* C* vector sampled on each training image.

(7) $$f_{C}=\sqrt{\frac{\sum_{i=1}^{l_{c}}{\left(C_{i_{new}}-\bar{C_{i}}\right)}^{2}}{l_{c}}}$$

where

$C_{new}$ = a sampled *C* vector on a new echocardiogram.
$\bar{C}$ = the mean *C* vector of the training set.
$l_{c}$ = The size of the sampled $C_{new}$ vector (64×l).

With the improved ASM and ShapeNet training explained, the final step is to perform segmentation of a new image, as described in the following section.

### 3.4. Left Ventricle Contour Reconstruction and Segmentation

**Figure 8:**
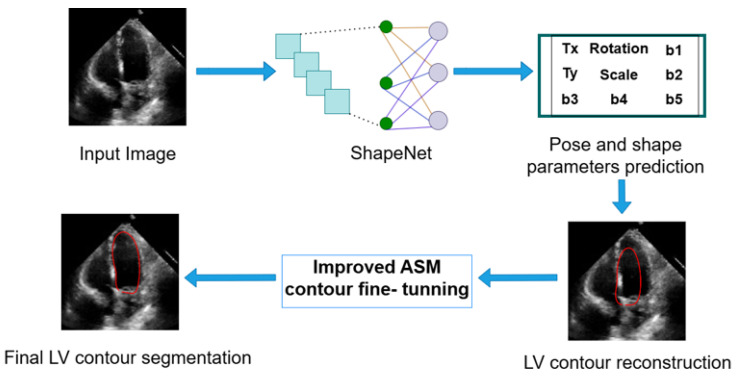
Complete inference pipeline of the ShapeNet+ASM approach.

ShapeNet predicts the optimal pose and shape parameters, including rotation (θ), translation ($T_{x},T_{y}$), and scale (σ), specific to the new image. Using these parameters, the left ventricle contour is reconstructed based on Equations (1) and (2). Once the shape predicted has been reconstructed, this contour acts as an automatic initialization for the improved ASM and will undergo fine-tuning to obtain the final LV contour. Figure 8 depicts the segmentation workflow described.

## 4. Results

In this section, we present the experiments and results of the ShapeNet and the improved ASM method, which were conducted on a server running on the Ubuntu operating system, with 32 GB of RAM. Additionally, two GPUs were used in parallel: an NVIDIA Tesla K40c and a Tesla T4. Finally, all experiments were implemented in MATLAB R2022b.

### 4.1. Dataset

The dataset was divided into two parts: training and test. For the training set, we used the CAMUS database [3], comprising a total of 800 images, with 400 corresponding to systole and 400 to diastole end of cycle. Data augmentation was applied to this set of 800 images, including rotation, translation, scaling transformations, and the addition of acoustic shadows, following the work of [33], resulting in a total of 4800 training images. On the other hand, for the test set, 49 systole images and 49 diastole images were reserved from the CAMUS database. Additionally, images extracted by Guzman et al. [34] from the EchoNet Dynamic database [35] were also used for testing; of these images, 207 corresponded to end-systole and 210 to end-diastole. These images were preprocessed following the methodology in [34], which included rigorous frame selection to isolate end-systolic and end-diastolic phases using the dataset’s provided timestamps and quality control metrics, automated region-of-interest cropping centered on the left ventricle using landmark detection heuristics, and intensity normalization through min-max scaling of pixel values to [0, 1]. These preprocessed images were then resized to 256 × 256 pixels to match our network ensemble input dimensions.

**Figure 9:**
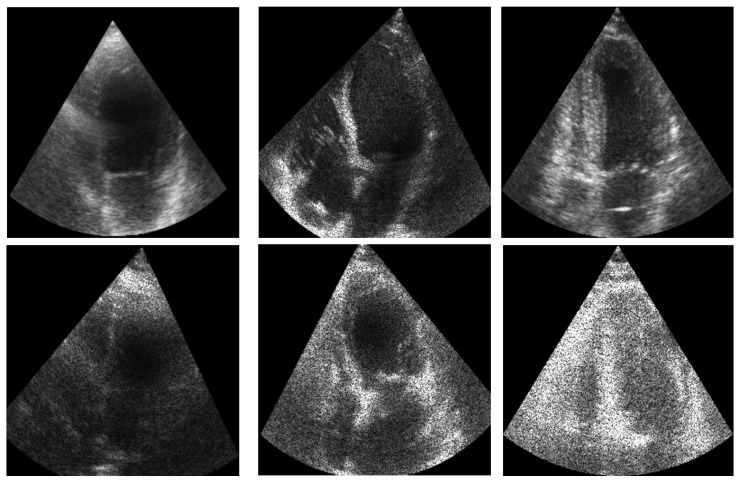
ShapeNet training images example.

Figure 9 illustrates an example of the images used for training the ShapeNet. The improved ASM was also trained with the set of 800 images extracted from the CAMUS database before the data augmentation process.

#### Data Augmentation Parameters

To increase the diversity of the training data and improve model generalization, the following augmentation techniques were applied to the original CAMUS dataset, resulting in 4800 training samples:

**Geometric Transformations**:

- Rotation: Limited to ±15°, to simulate minor probe orientation changes during acquisition.
- Translation: Random shifts of ±10 pixels along both X and Y axes, for natural LV positioning differences across patients.
- Scaling: Random scaling factors between 0.8× and 1.2×, simulating heart size variations.

**Ultrasound-Specific Augmentation**:

- Acoustic shadows: Simulated shadow artifacts were added through pixelwise multiplication of a spatial Gaussian kernel with selected image regions, following the methodology described in [33].

### 4.2. Evaluation of the Improved ASM

**Figure 10:**
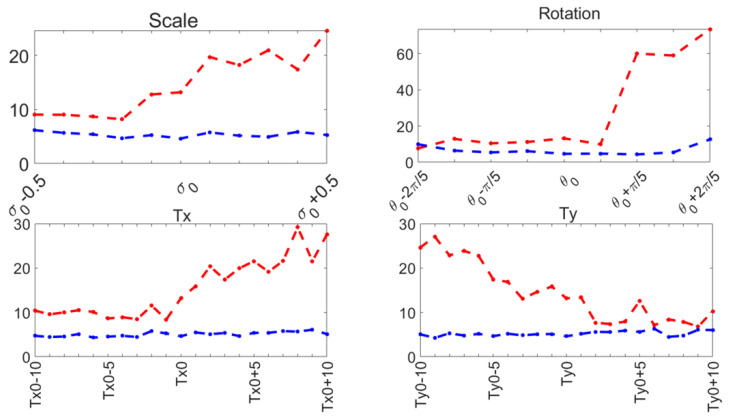
Capture range errors. Haussdorf distance: improved ASM (blue); original ASM [30] (red).

Capture range tests were performed on the ASM reported here (Section 3.3), and compared to the original ASM reported by Cootes [30]. The mean shape was manually aligned to the left ventricle on an US image, and it was automatically adjusted to the contour of the left ventricle using the original local search of the ASM and our function optimization method. This was repeated for a range of values around the initial manual pose values: $\sigma_{0},\theta_{0},Tx_{0},Ty_{0}$. The range of values for each pose parameter and the contour segmentation errors for each method are shown in Figure 10 for 30 diastole images.

The training parameters for both ASMs were as follows:

- 64 landmarks were used to represent the contour of the LV, as depicted in Figure 2.
- The length of the perpendicular sampled gray profiles for each training example of the PDM was 24 pixels.
- The explained shape variance was 90% for 5 principal components.

These parameters have been demonstrated to accurately represent the LV contour in previous works, as shown in [29,36]. During our capture range experiments, the original ASM reported in [30] failed to converge in several cases, causing run-time errors. Table 1 reports the number of capture range tests conducted and the number of run-time errors that occurred for 30 diastole images from the CAMUS database.

### 4.3. ShapeNet Contour Prediction Results

**Figure 11:**
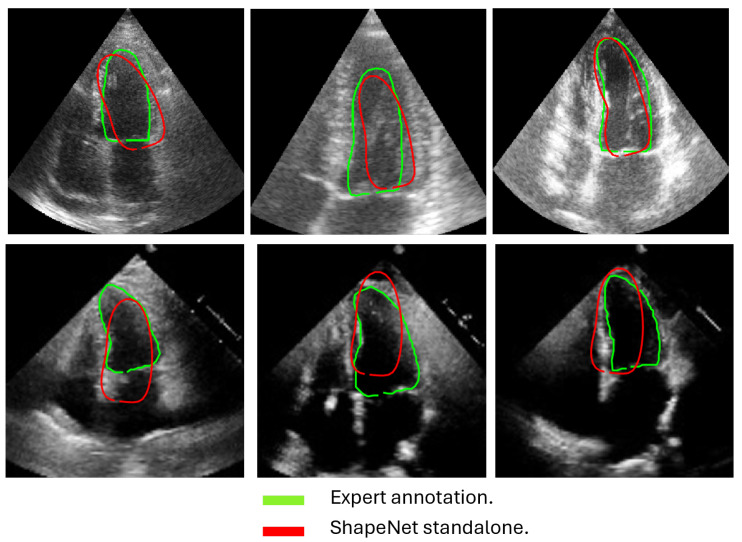
ShapeNet standalone systole segmentation examples for CAMUS (**top**) and EchoNet (**bottom**) images.

**Figure 12:**
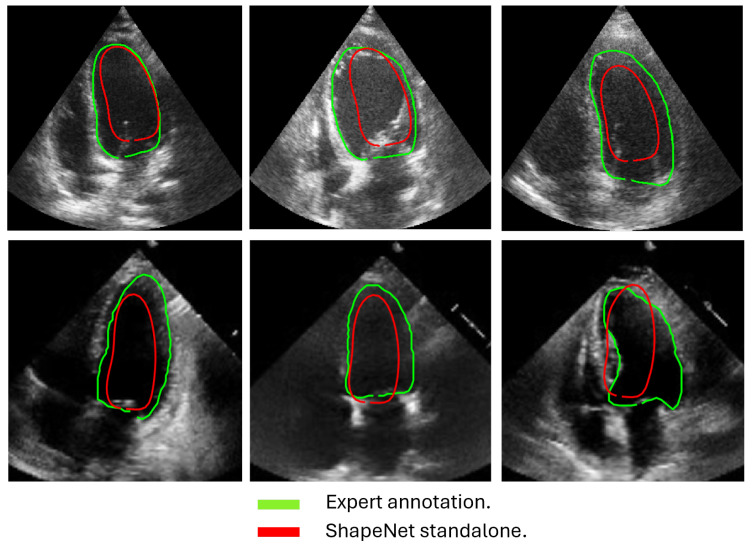
ShapeNet standalone diastole segmentation examples for CAMUS (**top**) and EchoNet (**bottom**) images.

In this section, we present the results obtained from the reconstruction of the LV using the parameters predicted by ShapeNet, following the algorithm shown in Figure 8. To evaluate these results, we used the Dice coefficient and Hausdorff distance (HD) expressed in pixels (px), compared against expert annotations. Table 2 shows the Dice and Hausdorff values for the two test datasets: EchoNet and CAMUS. In Figure 11 and Figure 12 are shown six examples of LV reconstruction for systole and diastole, respectively.

### 4.4. Improved ASM with ShapeNet Initialization

**Figure 13:**
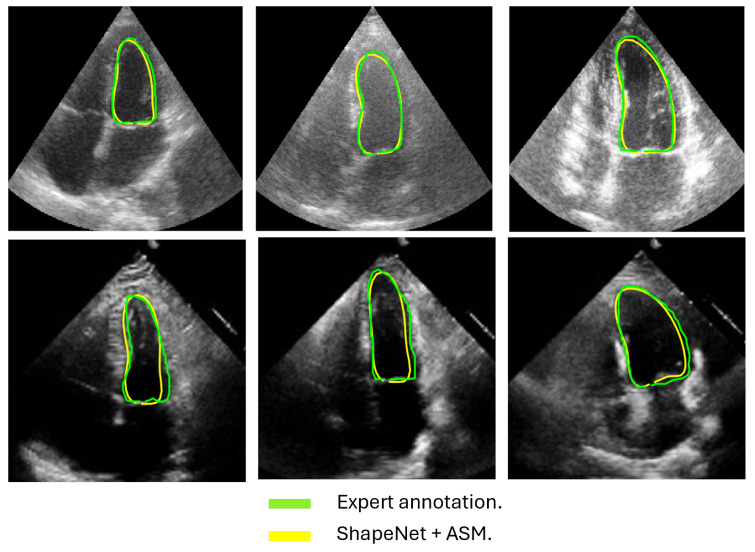
ShapeNet + improved ASM systole segmentation examples for CAMUS (**top**) and EchoNet (**bottom**) images.

**Figure 14:**
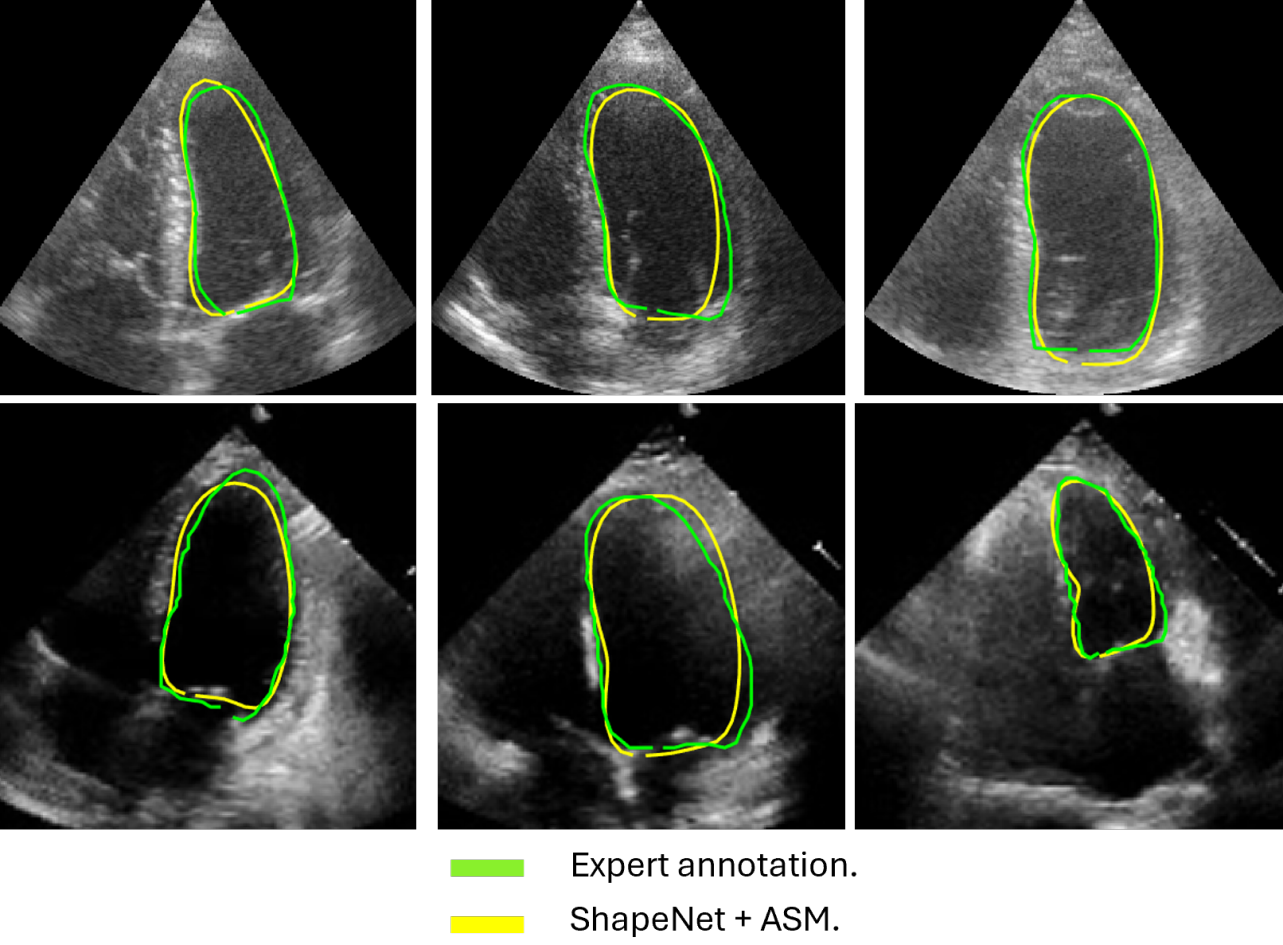
ShapeNet + improved ASM diastole segmentation examples for CAMUS (**top**) and EchoNet (**bottom**) images.

Following the algorithm depicted in Figure 8, the contour predicted by ShapeNet was used to automatically initialize the ASM described in Section 3.3. The result of combining these two methods can be observed in Figure 13 and Figure 14 for systole and diastole, respectively. In Table 3 is shown the Dice coefficient and Hausdorff distance for each of the test sets (CAMUS and EchoNet), with overall values of 0.83 ±0.09 for Dice and 7.36 ±8.02 pixels for Hausdorff distance.

### 4.5. ShapeNet + ASM Algorithm vs. Other Methods

This section provides a comparative analysis between the ShapeNet ensemble and two alternative segmentation approaches: (1) shape-based methods (Table 4) and (2) an in-house U-Net (Table 5). In Table 4, we compare the performance of our approach against other shape-based methods reported in [3] and in [26] trained and tested on the same dataset. On the other hand, we developed an in-house U-Net under the same experimental conditions as the ShapeNet. The in-house U-Net was configured with a batch size of 32 images, using the Adam optimizer with a learning rate of $1\times 10^{-3}$ for 50 training epochs, and the same dataset as described above was used to train both the U-Net and ShapeNet models. Additionally, the performance of this U-Net was evaluated using CAMUS and the EchoNet dataset, a fully independent dataset not previously seen by either the ShapeNet or the U-Net model. This setup ensured consistent conditions, for a fair comparison between the U-Net and ShapeNet methods. The performance metrics for each approach are shown in Table 4 and Table 5. We also conducted a statistical significance T-test on the Dice and HD values for ShapeNet + ASM and the in-house U-Net using the EchoNet database, as reported in Table 6.

## 5. Discussion

This study demonstrated that the proposed ShapeNet + ASM method achieved robust and competitive segmentation performance for left ventricle (LV) contours in echocardiography. Our results, evaluated across the CAMUS and EchoNet datasets, highlighted the method’s capability to generate statistically valid and anatomically accurate LV contours. The use of two datasets allowed us to employ the majority of the available images in the CAMUS dataset, to maximize the number of training patterns. We initially reserved a limited number of images to test our algorithm. To further test the accuracy of our proposal we used an independent unseen dataset, EchoNet Dynamic, which comprises a total of 417 images spanning both systole and diastole. As expected, the accuracy of ventricle segmentation was higher for the small test set CAMUS, and slightly lower for the independent test set EchoNet (Table 2, Table 3 and Table 5), which provides more representative values for accuracy than can be expected during clinical use.

The approach implemented in ShapeNet, where the parameters of a statistical shape model of the organ of interest are optimized with a convolutional neural network, provides restrictions that contribute to the explicability of the final segmentation results. All shapes produced were statistically valid organ shapes. As observed in Figure 11 and Figure 12, the results were always smoothed ventricle shapes located closely to the LV in the echocardiography, with a scale and rotation approximate to the expert annotation. However, for deformations, in some cases, the predicted values of the deformation vector *b* were not as accurate, which was reflected in a higher Hausdorff distance (see Table 2).

Our proposed ASM was used to improve the accuracy of the final segmentation of the LV. This ASM proved to be more accurate than the original ASM reported in [30]. In our capture range tests (see Figure 10), the improved ASM produced smaller mean values for the Hausdorff distance. Additionally, in some cases, when the initialization pose values were far from the ventricle contour, the original ASM failed to converge, causing run-time errors when the LV model grew outside the image. Table 1 shows that, for the improved ASM, the number of run-time errors was exceptionally low compared to the ASM reported in [30]. The improvements in accuracy and robustness of our ASM are most likely due to the use of all the gray level profiles concatenated in a single vector, as well as the objective function, which together provide the means to evaluate the image fitting of a whole ventricle contour, instead of the local adjustment point-by-point performed in the original ASM, and this was reflected in the final segmentation of the LV for the EchoNet dataset, as seen in Table 5 and in Figure 13 and Figure 14.

Table 4 presents a comparison of our results against the methods proposed in [3], specifically the BEASM approaches, the fully automatic BEASM (BEASM-fully) and the semi-automatic BEASM (BEASM-semi), which is manually initialized at three points: two at the LV base and one at the apex, alongside the ACNN method [26], which incorporates shape constraints. ShapeNet + improved ASM, being a fully automatic method, demonstrated competitive performance. Although BEASM-semi and ACNN achieved slightly higher Dice scores, the results in Table 4 indicate that our method achieved the lowest Hausdorff distance for both systole and diastole phases, demonstrating improved characterization of the LV contour over the methods listed in Table 4.

**Figure 15:**
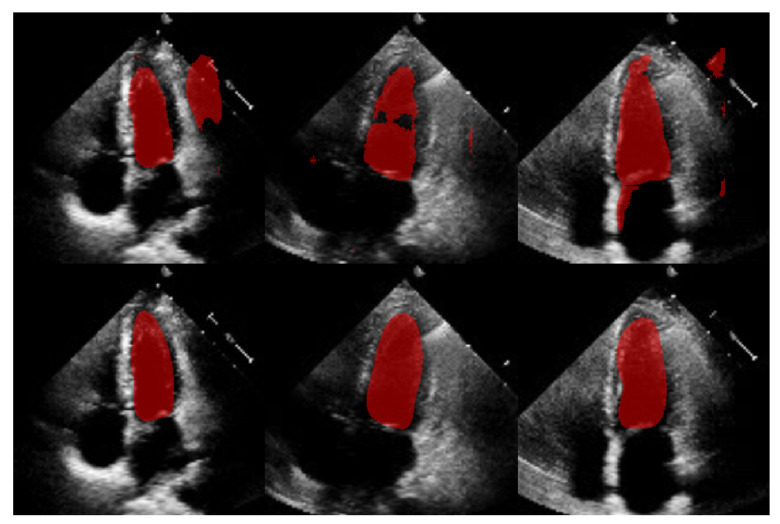
Comparison of segmentation masks between in-house U-Net (**top**) and ShapeNet + improved ASM (**bottom**) on EchoNet database.

The comparison with our in-house U-Net highlights that our method achieved competitive Dice scores and lower Hausdorff distances under the same training conditions (see Table 5). Specifically, the statistical analysis in Table 6 shows that for Dice scores, our method exhibited statistically significant improvements over the in-house U-Net. In contrast, the Hausdorff distance results reveal large effect sizes of d=9.31 for systole and d=14.132 for diastole, meaning a substantial reduction in spatial errors was achieved by our approach. These large effect sizes support the fact that our method produced a statistically valid LV contour closely aligned with expert annotations, demonstrating the robustness of our approach, even with a limited training set and the challenges of ultrasound imaging, particularly the presence of speckle noise. The added advantage of statistical shape constraints in our method reduced misclassified regions more effectively than traditional semantic classification, as depicted in Figure 15. While our method may not always have reached peak precision, it consistently achieved better segmentation performance on an entirely new dataset (EchoNet), as demonstrated in Table 5.

**Figure 16:**
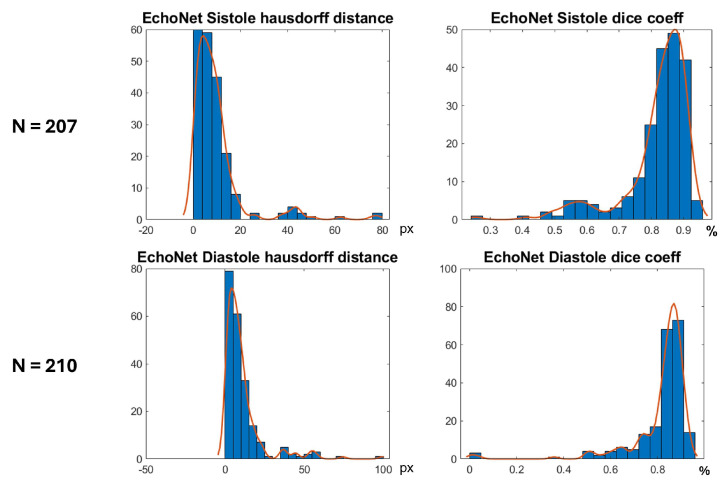
Segmentation errors (Dice score and HD) for EchoNet dataset using ShapeNet + improved ASM approach.

In Figure 16 are shown histograms of the ShapeNet + improved ASM approach for the Dice coefficient and Hausdorff distance values for systole and diastole for the EchoNet dataset. It can be observed that 75.84% and 77.61% of the cases for systole and diastole, respectively, fell within the range of 0.8 to 1. Similarly, for the Hausdorff distance, the majority of values were concentrated between 0 and 10 pixels, 68.11% and 66.66%, for systole and diastole, respectively. The density concentrated in the previously mentioned values demonstrate that our algorithm performed well and that the results were consistent, even with a completely new dataset, as was the case with EchoNet, in contrast to the methods shown in Table 4, which were trained and tested on images from the same dataset.

Despite the strengths of the proposed method, some limitations should be acknowledged. The CAMUS dataset, although it was augmented to 4800 images, remains relatively small for deep learning applications, and this may have affected the model’s generalization. In addition, the ShapeNet + improved ASM automatic initialization introduces a dependency that may reduce accuracy if there are significant deviations in initial conditions, such as patient positioning or heart orientation. The effectiveness of the improved ASM remains dependent on ShapeNet’s initialization quality. While ShapeNet’s ensemble architecture and the PDM’s shape constraints help mitigate this dependency, extreme imaging artifacts or anatomical anomalies may still lead to suboptimal ASM convergence. This limitation was evidenced in the EchoNet HD variance (10.21 px), where acoustic shadowing occasionally compromised initialization (Figure 12, bottom right). Nevertheless, our experimental results demonstrated that this combined approach remains clinically viable. ShapeNet’s parameter regression achieved Dice scores superior to 0.65, even in challenging cases (Table 2), providing sufficiently accurate initialization for ASM refinement. Furthermore, the ASM’s global objective function effectively corrected local errors when initialization was imperfect, as demonstrated by its superior performance compared to the original ASM (Figure 10).

On the other hand, the model’s reliance on a convolutional neural network ensemble and active shape models demands substantial computational resources for both training and inference. Each network in the ShapeNet ensemble was trained for a maximum of 50 epochs with early stopping (validation patience = 40 iterations). With our hardware configuration (NVIDIA Tesla K40c/T4), the average training time per epoch was approximately 20 min for each specialized CNN (rotation, translation, scale, and shape parameters). For comparison, our in-house U-Net implementation reached early stopping at 38 epochs with longer epoch durations of 41 min on average. Although we tried to make it a lightweight model, this requirement may limit accessibility and real-time application in clinical settings without the availability of modern GPUs. Regarding this issue, we conducted performance tests on a current gaming computer with the following specifications: 24 GB of RAM, an 8th generation Core i7 processor, and an NVIDIA GeForce GTX 1060 with Max Q with 6 GB, achieving offline segmentation of 207 LV images in an average of 18 min (5.27 s per image).

Concerning the morphology of the ventricle, although ShapeNet generally performs well, the segmentation accuracy during the systolic phase may be compromised due to increased left ventricular deformation during contraction, along with greater deformation observed in the diastolic phase, as indicated by higher Hausdorff distances in some cases. Our method showed enhanced robustness with limited training data, offering a statistically grounded alternative to traditional segmentation models.

Future work will involve exploring the incorporation of additional datasets to further enhance the model’s inference capabilities. Additionally, we plan to investigate techniques such as model pruning and knowledge distillation to reduce computational demands, while preserving the performance advantages of the ensemble.

## 6. Conclusions

In this paper, we proposed a new scheme for automatic LV segmentation in echocardiography images. The proposal consists of two stages: ShapeNet, which is an ensemble of CNNs to predict pose and shape parameters; and an improved ASM, which is initialized with the parameters estimated by the ensemble of neural networks, in order to fine-tune the LV contours for improved segmentation. Our study demonstrated that integrating ShapeNet with an improved ASM enhanced LV segmentation accuracy and effectively prevented blob artifacts commonly found in semantic segmentation. Our algorithm was tested on two different datasets, CAMUS and EchoNet, providing an overall Dice coefficient of 0.83 and a Hausdorff distance of 7.36 pixels for both systole and diastole.

A major strength of our approach is its ability to automatically generate statistically valid shapes, offering a new perspective on the utilization of convolutional neural networks (CNNs) in medical imaging. When combined with the improved ASM, which outperformed traditional ASM techniques, our method provided competitive and accurate fitting of the LV contour compared to existing shape-based methods, as evidenced by higher Dice coefficients and lower Hausdorff distances in ultrasound images. Notably, we demonstrated that the ShapeNet + ASM approach was more robust with a limited training set than traditional semantic segmentation methods such as U-Net under the same conditions. Despite these strengths, limitations should be considered, including the need for substantial computational resources, the limited availability of training images, and the complexity of ultrasound images, due to speckle noise and heart morphology variations in both systolic and diastolic phases.

The ShapeNet and improved ASM methodologies presented here offer a promising alternative to semantic segmentation CNNs in medical image analysis. This approach, based on statistically valid shape adjustment, holds potential for broad applications in automated medical image analysis and clinical decision-making.

## Figures and Tables

**Table 1:** Run-time errors for ASM [30] and improved ASM.

Type of Capture Range Test				
	**Rotation**	**Tx**	**Ty**	**Scale**
Number of tests performed	270	630	630	330
Percentage of ASM run-time errors	17.8	3.8	2.2	15.5
Percentage of improved ASM run-time errors	0.7	0.0	0.0	0.6

**Table 2:** ShapeNet standalone segmentation errors on CAMUS vs. EchoNet (independent test set).

EchoNet			
Number of evaluated images	Cycle	Mean Dice	Mean HD (px)
207	Systole	0.65 ± 0.11	30.24 ± 9.87
210	Diastole	0.62 ± 0.10	37.07 ± 13.0
**CAMUS**			
Number of evaluated images	Cycle	Mean Dice	Mean HD (px)
49	Systole	0.76 ± 0.10	19.13 ± 7.91
49	Diastole	0.74 ± 0.10	25.86 ± 14.04

**Table 3:** ShapeNet + improved ASM segmentation errors on CAMUS vs. EchoNet (independent test set).

EchoNet			
Number of evaluated images	Cycle	Mean Dice	Mean HD (px)
207	Systole	0.81 ±0.10	9.72 ±11.66
210	Diastole	0.81 ±0.13	10.70 ±13.21
**CAMUS**			
Number of evaluated images	Cycle	Mean Dice	Mean HD (px)
49	Systole	0.87 ±0.07	4.26 ±3.29
49	Diastole	0.87 ±0.07	4.88 ±4.65
**Overall performance**			
Cycle	Overall Dice		Overall HD (px)
Systole	0.84 ±0.08		6.99 ±7.47
Diastole	0.84 ±0.10		7.79 ±8.93

**Table 4:** Comparison between ShapeNet + improved ASM and other shape-based methods trained and tested with the same dataset.

Method	Database	Systole		Diastole	
		**Mean Dice**	**Mean HD (px)**	**Mean Dice**	**Mean HD (px)**
ShapeNet + improved ASM	CAMUS	0.875 ±0.07	4.26 ±3.29	0.870 ±0.07	4.88 ±4.65
BEASM-Fully [3]	CAMUS	0.826 ±0.09	9.9 ±5.1	0.879 ±0.065	9.2 ±4.9
BEASM- Semi [3]	CAMUS	0.861 ±0.07	7.7 ±3.2	0.920 ±0.03	6.0 ±2.4
ACNN [26]	CETUS’14	0.873 ±0.05	7.75 ±2.65	0.912 ±0.023	6.96 ±1.75

**Table 5:** Performance comparison between ShapeNet + improved ASM and in-house U-Net on CAMUS vs. EchoNet (independent test set).

CAMUS				
**Method**	**Systole**		**Diastole**	
	Mean Dice	Mean HD (px)	Mean Dice	Mean HD (px)
ShapeNet + improved ASM	0.87 ±0.07	4.26 ±3.29	0.87 ±0.07	4.88 ±4.65
In-house U-Net	0.90 ±0.06	9.64 ±7.36	0.93 ±0.03	12.44 ±12.38
**EchoNet**				
	Mean Dice	Mean HD (px)	Mean Dice	Mean HD (px)
ShapeNet + improved ASM	0.81 ±0.10	9.72 ±11.66	0.81 ±0.13	10.70 ±13.21
In-house U-Net	0.75 ±0.09	19.03 ±8.44	0.78 ±0.10	24.81 ±16.19

**Table 6:** *T*-Test results for ShapeNet + ASM vs. U-Net using EchoNet database.

	Dice Score		HD	
	Systole	Diastole	Systole	Diastole
*p* value	$p<1\times 10^{-08}$	$p<1^{-02}$	$p<1\times 10^{-16}$	$p<1\times 10^{-17}$
*h* value	1.0	1.0	1.0	1.0
Effect size (d)	0.0607	0.0321	9.31	14.132

## Data Availability

CAMUS database can be found at https://www.creatis.insa-lyon.fr/Challenge/camus/ (accessed on 10 January 2021). In addition, the EchoNet Dynamic database can be downloaded from https://echonet.github.io/dynamic/ (accessed on 22 June 2023).

## Abbreviations

The following abbreviations are used in this manuscript:

LV	Left Ventricle
CNN	Convolutional Neural Network
PDM	Point Distribution Model
ASM	Active Shape Model
PCA	Principal Component Analysis
HD	Hausdorff Distance
SSM	Statistical Shape Model
px	Pixel
